# 1376. A Retrospective Analysis of Intravenous Fluid Therapy Practice at Hospital Admission for Adults with Dengue

**DOI:** 10.1093/ofid/ofad500.1213

**Published:** 2023-11-27

**Authors:** Yvonne M F Chia, James W J Kang, Felicia C Y Lea, Eng Eong Ooi, Jenny G Low

**Affiliations:** Singapore General Hospital, Singapore, Singapore; Singapore General Hospital, Singapore, Singapore; Singapore General Hospital, Singapore, Singapore; Duke-NUS Medical School, Singapore, Not Applicable, Singapore; Singapore General Hospital, Singapore, Singapore

## Abstract

**Background:**

Dengue epidemics in Asia was almost exclusively a childhood disease but recently dengue in adults have become increasingly prevalent. Yet intravenous fluid replacement (IVFR), the main supportive therapy for dengue, was developed for pediatric dengue. Evidence-based guidance for fluid replacement, especially for older adults with concomitant chronic diseases that both increases risk of severe dengue and complicate fluid replacement, is lacking. We retrospectively analyzed the use of IVFR in adult dengue patients at admission to a tertiary hospital.

**Methods:**

Medical records of patients diagnosed with dengue (confirmed by rapid NS1 kit or laboratory confirmation of RT-PCR, NS1 or IgM) from January 2020 to December 2022 were analyzed. Recovery phase was defined as fever defervescence for >12 hours with rising platelet count. Appropriateness of IVFR was based on disease severity according to the 2009 WHO dengue classification scheme.

**Results:**

Preliminary analysis of the first 30 patients found an almost equal number of males (47%) and females. The median age was 52.5 years (range 15.0-83.0). 20 patients had at least one comorbidity with hyperlipidemia (33%), hypertension (23%) and ischemic heart disease (10%) being the most common. Average time from onset to presentation was 4.3 days (95% CI: 3.8-4.9). 16 (53%) met the WHO classification scheme as requiring IVFR at admission; 4 hypotension, 5 acute kidney injury (AKI), 2 hypotension with AKI, 4 hemoconcentration, 1 AKI with hemoconcentration. Of these 16, one also had altered mental status and another had hemophagocytic lymphohistiocytosis-like presentation. Despite 47% having uncomplicated dengue, all patients had IVFR at admission. Total fluid volume administered during hospitalization varied across the population and fluctuated daily for each patient, with 8 patients receiving IVFR beyond the recovery phase. One developed fluid overload while another had IV cannula-related thrombophlebitis. There was no mortality.

**Conclusion:**

Preliminary findings of our on-going study suggest excessive use of IVFR and the lack of standardization possibly contributed to two iatrogenic complications. We call for development of evidence-based protocol on fluid management in older dengue patients.

**Disclosures:**

**All Authors**: No reported disclosures

